# mTOR Regulation of N-Myc Downstream Regulated 1 (NDRG1) Phosphorylation in Clear Cell Renal Cell Carcinoma

**DOI:** 10.3390/ijms24119364

**Published:** 2023-05-27

**Authors:** Anisha Valluri, Jessica Wellman, Chelsea L. McCallister, Kathleen C. Brown, Logan Lawrence, Rebecca Russell, James Jensen, James Denvir, Monica A. Valentovic, Krista L. Denning, Travis B. Salisbury

**Affiliations:** 1 Department of Biomedical Sciences, Joan C. Edwards School of Medicine, Marshall University, 1 John Marshall Drive, Huntington, WV 25755, USA; valluri4@live.marshall.edu (A.V.); wellman88@live.marshall.edu (J.W.); thompsonch@marshall.edu (C.L.M.); brown364@marshall.edu (K.C.B.); denvir@marshall.edu (J.D.); valentov@marshall.edu (M.A.V.); 2 Cabell Huntington Hospital Laboratory, Department of Pathology, Joan C. Edwards School of Medicine, Marshall University, Huntington, WV 25701, USA; loganlawrence38@gmail.com (L.L.); rebecca.russell@chhi.org (R.R.); haught5@marshall.edu (K.L.D.); 3 Edwards Comprehensive Cancer Center, Department of Oncology, Joan C. Edwards School of Medicine, Marshall University, Huntington, WV 25701, USA; jensenj@marshall.edu

**Keywords:** N-Myc Downstream Regulated 1, NDRG1, clear cell renal cell carcinoma, mTOR, mTORC1, mTORC2, proteomics

## Abstract

The mechanistic target of rapamycin (mTOR) kinase is a component of two signaling complexes that are known as mTOR complex 1 (mTORC1) and mTORC2. We sought to identify mTOR-phosphorylated proteins that are differently expressed in clinically resected clear cell renal cell carcinoma (ccRCC) relative to pair-matched normal renal tissue. Using a proteomic array, we found N-Myc Downstream Regulated 1 (NDRG1) showed the greatest increase (3.3-fold) in phosphorylation (on Thr346) in ccRCC. This was associated with an increase in total NDRG1. RICTOR is a required subunit in mTORC2, and its knockdown decreased total and phospho-NDRG1 (Thr346) but not NDRG1 mRNA. The dual mTORC1/2 inhibitor, Torin 2, significantly reduced (by ~100%) phospho-NDRG1 (Thr346). Rapamycin is a selective mTORC1 inhibitor that had no effect on the levels of total NDRG1 or phospho-NDRG1 (Thr346). The reduction in phospho-NDRG1 (Thr346) due to the inhibition of mTORC2 corresponded with a decrease in the percentage of live cells, which was correlated with an increase in apoptosis. Rapamycin had no effect on ccRCC cell viability. Collectively, these data show that mTORC2 mediates the phosphorylation of NDRG1 (Thr346) in ccRCC. We hypothesize that RICTOR and mTORC2-mediated phosphorylation of NDRG1 (Thr346) promotes the viability of ccRCC cells.

## 1. Introduction

In the United States, renal cell carcinoma (RCC) is the sixth and tenth most common cancer in men and women, respectively [1,2,3]. Clear cell renal cancer carcinoma (ccRCC) is the most common renal cancer subtype and represents approximately 80% of all renal cancers [1,2,3,4]. If early-stage ccRCC is diagnosed and is still localized to the kidney, it can be treated with surgical resection [1,2,3,4]. However, the insidious growth of this cancer allows tumors to progress undetected toward late-stage disease [1,2,3,4]. Consequently, approximately 20% of patients will be diagnosed with late-stage metastatic ccRCC [1,2]. The five-year survival rate drops from 80% to less than 20% for early-stage versus late-stage ccRCC, respectively [1,2,3]. Thus, early detection and new therapies are critical for ccRCC.

Men have a two-fold higher incidence rate for renal cell carcinoma than women [5,6,7]. The prognosis for renal cell carcinoma is also worse for men than women [5,6,7]. The incidence and mortality rates of renal cancer are also influenced by cigarette smoking, obesity, hypertension, and race [5,6,7,8,9]. 

The phosphoinositide 3 kinase (PI3K)/AKT/mechanistic Target Of Rapamycin (mTOR) pathway promotes cell growth, cell survival, proliferation, and tumor growth [10,11,12]. mTOR mediates these effects as the central kinase of two protein complexes known as mTOR complex 1 (mTORC1) and mTOR complex 2 (mTORC2) [11,12,13]. Growth factors and nutrients stimulate mTORC1 signaling [14,15,16]. Growth factors via activation of PI3K induce mTORC2 activity [12,17]. mTORC1 consists of six different proteins, including mTOR [14,15]. The complex mTORC2 consists of mTOR along with six other distinct proteins [15,17]. RAPTOR is a protein that is present in mTORC1 but not mTORC2 [15,18]. RICTOR is a protein that is part of mTORC2 but not mTORC1. RICTOR is required for mTORC2 activity [15,19]. 

The tumor suppressor Von Hippel–Lindau (VHL) is inactivated in approximately 80% of ccRCC cases [20,21,22]. The inactivation/loss of VHL stabilizes Hypoxia Inducible Factor 1 subunit alpha (HIF-1α) protein [23,24,25]. HIF-1α promotes vascular endothelial-derived growth factor (VEGF) expression and, in turn, VEGF signaling [26]. Pathways induced by VEGF include mTOR [27]. Genetic alterations in at least one gene that can be linked to potential changes in mTOR signaling occur in approximately 20% of ccRCC cases [21]. Drugs that inhibit mTORC1 (such as everolimus and temsirolimus) are FDA-approved for the treatment of advanced ccRCC [4,28]. Overall, however, the mTORC1 inhibitors have limited efficacy in ccRCC and have side effects [29,30,31]. Upon activation, mTOR phosphorylates specific downstream proteins that are specifically linked to mTORC1 or mTORC2 signaling [15,17,32]. We sought to identify mTOR-regulated downstream protein targets that are differentially phosphorylated in clinically resected ccRCC samples relative to pair-matched normal renal tissue controls. 

To this end, we used a cancer-specific proteomic array to simultaneously detect approximately 440 cancer-associated proteins and phospho-proteins in ccRCC tumors and their pair-matched normal renal tissue controls (N = 22). This array contains probes for proteins that are phosphorylated by mTORC1 and mTORC2 [33]. The results showed that the level of mTORC1 signaling in ccRCC was not different from its levels in pair-matched normal renal tissue, given that the phosphorylation of primary mTORC1 targets (phospho-p70 S6 Kinase (Thr389), phospho-S6 (S235/236), and phospho-4E-BP1 (Thr37/46)) was not significantly increased in ccRCC samples relative to normal renal tissue. The phosphorylation of NDRG1 (on Thr346) showed the greatest statistically significant increase in ccRCC compared with noncancerous renal tissue. Further study showed the phosphorylation of NDRG1 (Thr346) in ccRCC cells was maintained by RICTOR and mTOR in complex 2. Treatments that inhibit RICTOR and mTORC2 reduce phospho-NDRG1 (Thr346) and cancer cell viability. Collectively, these data show RICTOR and mTOR in complex 2 maintain the phosphorylation of NDRG1 (Thr346) in ccRCC. We hypothesize that phospho-NDRG1 (Thr346) promotes the survival of cancer cells, and thus its targeting could potentially be efficacious for ccRCC.

## 2. Results

### 2.1. Differentially Expressed Proteins and Phospho-Proteins in ccRCC

To characterize changes in signaling at the level of proteins and phospho-proteins in ccRCC, we carried out protein array studies using Reverse Phase Protein Array (RPPA) technology to measure the expression of approximately 440 cancer-associated proteins and phospho-proteins. Twenty-two clinically resected ccRCC samples and their 22 pair-matched normal renal tissue controls were compared. Kidney samples were obtained from treatment naïve male patients. Men are more prone (two-fold) to ccRCC, and the vast majority of the participants were male, which is why we chose to study 22 male samples in this study [5,6,7]. The median age at diagnosis was 57 years. The Body Mass Index (BMI) was greater than 30 for all patients, except for one patient who had a BMI of 29. Based on Fuhrman nuclear grading, three samples were grade I, 11 were grade II, four were grade III, and four were grade IV tumors. We identified 39 unphosphorylated and eight phosphorylated proteins, of which 20 were upregulated and 27 were downregulated by at least 50% in ccRCC relative to pair-matched normal renal tissue (Table 1).

We identified statistically significant changes in protein expression that correspond with changes in cellular metabolism. Mitochondrial proteins (Mitochondrial Encoded Cytochrome C Oxidase, Succinate Dehydrogenase Complex Iron–Sulfur Subunit B, Cytochrome C Oxidase Subunit 4l1, and Succinate Dehydrogenase Complex Flavoprotein Subunit A) were significantly (*p* < 0.05) reduced, and proteins involved in glycolysis (Hexokinase 2, Pyruvate Kinase, and Lactate Dehydrogenase) were significantly (*p* < 0.05) increased in ccRCC compared with non-cancerous renal tissue (Table 1). NDRG1 showed the greatest fold increase (3.3-fold) in phosphorylation (on Thr346) in ccRCC relative to normal renal tissue (Table 1). Considering that the phosphorylation of primary mTORC1 downstream targets was not different in ccRCC samples relative to pair-matched normal renal tissue, we focused on the regulation of NDRG1 phosphorylation on Thr346 in ccRCC cancer cells.

### 2.2. Total NDRG1 Protein and Phospho-NDRG1 (Thr346) Are Increased in ccRCC

**Figure 1:**
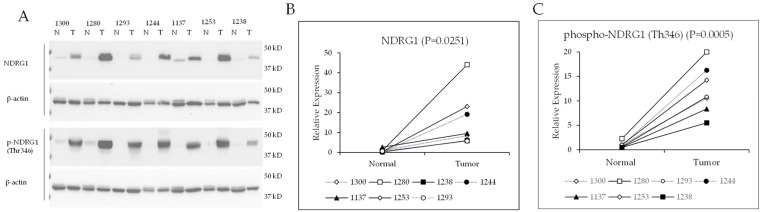
Total and phosphorylated NDRG1 (Thr346) are increased in ccRCC samples. (**A**) Western blots for total NDRG1, phospho-NDRG1 (Thr346), and β-actin in ccRCC tumor (T) samples pair-matched with normal (N) renal tissue. Quantification of (**B**) NDRG1 and (**C**) phosphorylated NDRG1 (Thr346) western blot data. Differences between ccRCC and normal tissue protein expression (N = 7) were analyzed using paired *t*-tests.

Western blot experiments were performed to validate the protein array data. The results showed the level of total NDRG1 protein and phosphorylated NDRG1 (Thr346) was significantly increased in renal tumors (T) compared to pair-matched normal (N) renal tissue (Figure 1). Notably, these ccRCC and pair-matched normal renal tissue samples (N = 7) were different from those used in the initial protein array study. These data show that the increase in total NDRG1 and phospho-NDRG1 (Thr346) occurs repeatably in ccRCC (Figure 1).

### 2.3. Reducing RICTOR Causes a Reduction in Total NDRG1 Protein and Phospho-NDRG1 (Thr346) in ccRCC Cells

**Figure 2:**
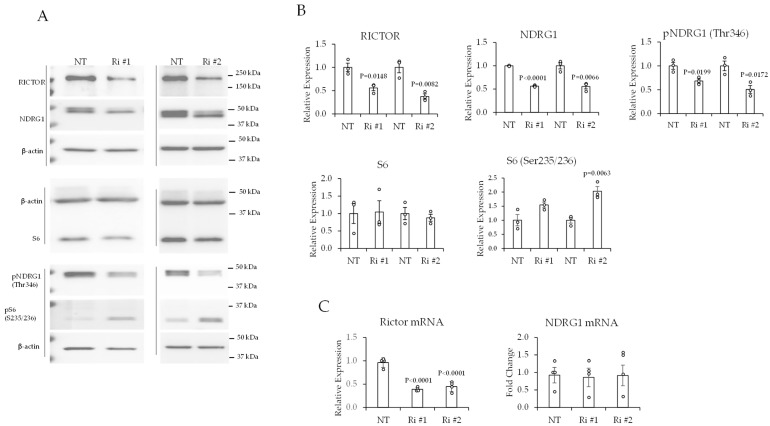
Short interfering RNA (siRNA)-mediated knockdown of RICTOR caused reductions in total NDRG1 protein and phosphorylated-NDRG1 (Thr346). (**A**) Western blot data of 786-0 cells transfected with siRNAs that were either non-targeting (NT) or specific for two different regions of RICTOR mRNA (Ri #1 and Ri #2). Cells were transfected for 96 h. (**B**) Quantification of western blot data. (**C**) Real-time PCR data of 786-0 cells transfected with indicated siRNAs for 96 h. Differences between control (NT) and Rictor knockdown (Ri #1, Ri #2) cells were analyzed by unpaired *t*-tests. Data are represented as the mean ± SEM (N = 3).

**Figure 3:**
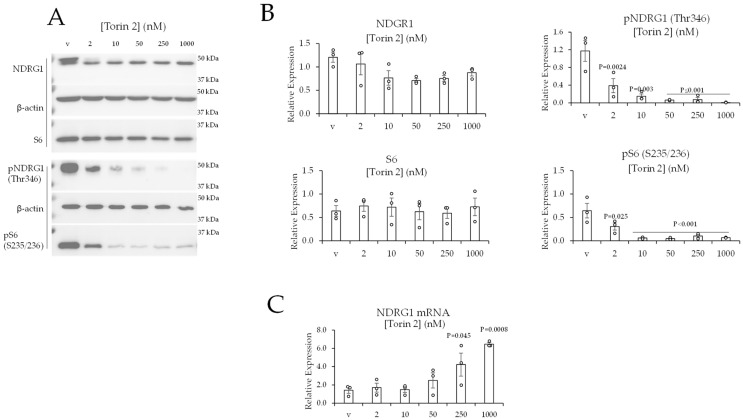
Torin 2 reduces the levels of phosphorylated NDRG1 (Thr346) in 786-0 cells. (**A**) Western blot data of 786-0 cells treated with vehicle or Torin 2 for 24 h. (**B**) Quantification of western blot data. (**C**) 786-0 cells were treated for 24 h, and mRNA expression was quantified by real-time PCR. Data are represented as the mean ± SEM (N = 3). Statistical analyses were performed using one-way ANOVA followed by Dunnett’s multiple comparison tests.

**Figure 4:**
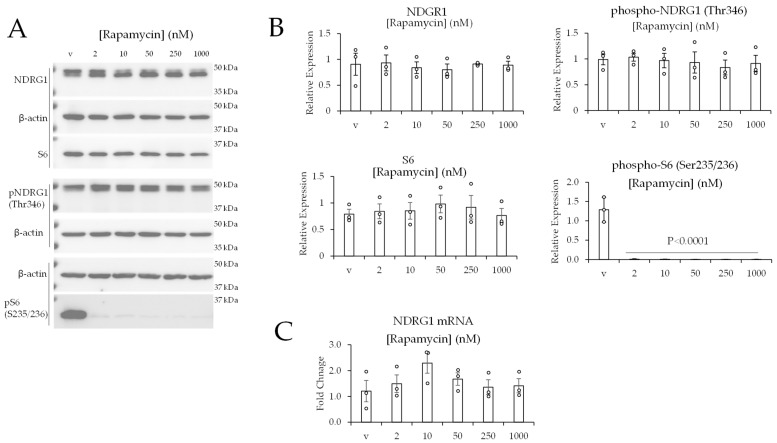
Inhibition of mTORC1 has no impact on the levels of NDRG1 or phospho-NDRG1 (Th346). (**A**) Western blot data of 786-0 cells treated with vehicle (v) or Torin 2 for 24 h. (**B**) Quantification of western blot data. (**C**) These 786-0 cells were treated for 24 h, and mRNA expression was quantified by real-time PCR. Data are represented as the mean ± SEM (N = 3). Statistical analyses were performed using one-way ANOVA followed by Dunnett’s multiple comparison tests.

In glioblastoma, mTORC2 regulates the phosphorylation of NDRG1 (on Thr346) [34]. To test whether NDRG1 is a mTORC2 target in ccRCC, we sought to selectively disrupt the activity of mTORC2 without perturbing the activity of mTORC1. To this end, we transiently transfected 786-0 ccRCC cells with short interfering RNA (siRNA) corresponding to RICTOR or non-targeting (NT) siRNA. The 786-0 cell line was chosen because it is defective in VHL expression and thus is a good cell line model of ccRCC, given that VHL is inactivated in approximately 80% of ccRCC cases [20,21,22,35]. The 786-0 cell line also expressed high levels of high phospho-NDRG1 (Thr346) and total NDRG1 (Figure 2, Figure 3 and Figure 4), which is consistent with the clinical samples (Figure 1). RICTOR is an obligatory subunit of mTORC2 that is not a component of mTORC1 [12,15]. We assessed two different RICTOR siRNAs (Ri #1 and Ri #2) that were designed to target different regions of RICTOR mRNA. Using this approach, we reduced RICTOR protein by 40% (Ri #1) and 60% (Ri #2) compared with control NT siRNA (Figure 2B). The decrease in RICTOR was associated with a statistically significant reduction in total NDRG1 protein (by ~50%) and phosphorylated NDRG1 (on Thr346) (between ~30 and 50%, for Ri #1 and Ri #2, respectively) relative to cells transfected with NT siRNA (Figure 2B). The interaction between mTORC1 and mTORC2 is dynamic [36], such that inhibition of mTORC2 can cause an increase in the activity of mTORC1, as shown by the statistically significant increase in the phosphorylation of S6 (S235/236), which is a readout of mTORC1 activity, in cells transfected with Ri # 2 relative to control cells transfected with NT-siRNA (Figure 2B). The increase in phospho-S6 (S235/236) in response to Ri #2 was not associated with an increase in total S6 protein (Figure 2B). The reduction in total NDRG1 protein in response to RICTOR knockdown was not associated with a decrease in the levels of NDRG1 mRNA (Figure 2C). These data suggest that RICTOR facilitates the phosphorylation of NDRG1 (Thr346) in part by promoting NDRG1 protein stability.

### 2.4. Concurrent Inhibition of mTOR in Complex 1 and Complex 2 Decreases the Levels of Phospho-NDRG1 (Thr346) in ccRCC Cells

To further investigate the regulation of NDRG1 by mTORC2, we treated 786-0 cells with increasing concentrations (2, 10, 50, 250, and 1000 nM) of Torin 2 for 24 h. Torin 2 is a recent mTOR inhibitor that concurrently inhibits mTOR in complex 1 and complex 2 [37]. At 2 nM, Torin 2 statistically significantly reduced the phosphorylation of NDRG1 at Thr346 by ~60% (Figure 3B). Torin 2 at higher concentrations induced greater reductions in phospho-NDRG1 (Thr346) (by ~85% at 10 nM, >90% at 50 and 250 nM, and complete suppression at 1000 nM) (Figure 3B). Torin 2 stimulated reductions in phospho-NDRG1 (Thr346) were not associated with reductions in total NDRG1 (Figure 3B). As shown by the western blot, Torin 2 induced a shift in the total NDRG1 band towards higher mobility, which is consistent with a decrease in the phosphorylation of NDRG1 (Figure 3A). As anticipated, because Torin 2 also inhibits mTOR in complex 1, the phosphorylation of the mTORC1 target, S6 (on S235/236), was significantly reduced in cells treated with Torin 2 (Figure 3). Maximal suppression of NDRG1 phosphorylation (Thr346) was correlated with a statistically significant increase in NDRG1 mRNA in response to 250 and 1000 nM Torin 2 treatments (Figure 3C).

#### Inhibition of mTORC1 Has No Impact on the Levels of Phospho-NDRG1 (Thr346)

We also assessed the effect of rapamycin on the phosphorylation of NDRG1 (on Thr346). Rapamycin is a potent inhibitor of mTORC1 that can partially inhibit mTORC2 after long-term exposure in some cell lines [36]. Rapamycin was applied to 786-0 cells at increasing concentrations (2, 10, 50, 250, and 100 nM) for 24 h. Western blot data showed rapamycin had no impact on the levels of total NDRG1 protein, phospho-NDRG1 (Thr346), or NDRG1 mRNA (Figure 4A–C). Rapamycin reduced the phosphorylation of the mTORC1 target phospho-S6 (S235/236) (Figure 4B). Given that Torin 2 is a potent inhibitor of both mTORC1 and mTORC2 that completely ablates the phosphorylation of NDRG1 (Thr346) (Figure 3), and that phospho-NDRG1 (Thr346) is resistant to rapamycin at all concentrations tested (Figure 4), these data indicate that mTOR in complex 2, but not complex 1, mediates the phosphorylation of NDRG1 (on Thr346) in ccRCC cells. 

#### 2.5. mTOR and RICTOR Promote the Viability of ccRCC Cells

**Figure 5:**
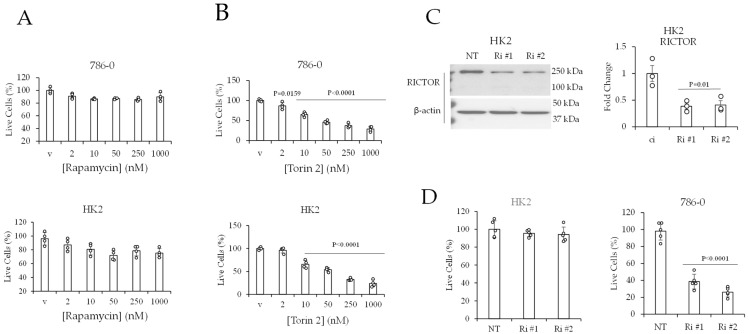
mTOR in complex 2 and RICTOR promote the viability of 786-0 cells. The % of viable cells after treatment with (**A**) rapamycin or (**B**) Torin 2 for 96 h. (**C**) Western blot data of HK2 cells transfected with short interfering RNAs (siRNA) that are either non-targeting (NT) or against two different regions of Rictor mRNA (Ri #1 and Ri #2) for 96 h. Quantification of Western blot data. (**D**) The % of viable cells after transfection of the indicated siRNAs for 3 days. The % cell viability data are represented as the mean ± SD of N = 4. The quantified western blot data shown is the mean ± SEM of N = 3. Statistical analyses were performed using one-way ANOVA followed by Dunnett’s multiple comparison tests.

Because mTOR promotes cell growth and survival, we hypothesized that mTOR is essential for ccRCC cell viability. We questioned if this mTOR effect is mediated through complex 1 or complex 2. We also probed whether the requirement for mTOR is higher in cancer cells than in non-cancerous cells. To test this hypothesis, 786-0 and HK2 cells were treated with rapamycin or Torin 2 at increasing (2, 10, 50, 250, and 1000 nM) concentrations for 3 days. The effect on cell viability was assessed by calcein fluorescence. The results showed that 786-0 and non-cancerous HK2 renal epithelial cells were resistant to the mTORC1 inhibitor rapamycin (Figure 5A). There was no change in the percentage of live cells in response to rapamycin at all tested concentrations (Figure 5A). Conversely, both cell lines were equally sensitive to Torin 2. In both cell lines, cell viability was significantly reduced by 35% at 10 nM, by 50% at 50 nM, and by 75% at 250 and 1000 nM in response to Torin 2 (Figure 5B).

Given that concurrent inhibition of mTORC1 and mTORC2 by Torin 2 non-selectively reduced the viability of HK2 and 786-0 cells, we tested whether selective inhibition of mTORC2 would reduce the viability of 786-0 cells without reducing the viability of HK2 cells. To this end, cells were transfected with RICTOR siRNAs (Ri #1 and Ri #2). As noted, RICTOR is a required subunit of mTORC2 [15,19]. The results showed that RICTOR siRNAs significantly reduced (by ~60%) the levels of RICTOR protein in HK2 cells (Figure 5C). This, however, did not affect the viability of HK2 cells (Figure 5D). We previously showed that the RICTOR siRNAs (Ri #1 and Ri #2) significantly reduced (by ~50%) the RICTOR protein in 786-0 cells (in Figure 2). This was associated with a significant reduction in 786-0 cell viability compared with 786-0 cells transfected with non-targeting control siRNA (Figure 5D).

##### Torin 2 Stimulates Apoptosis in 786-0 Cells

**Figure 6:**
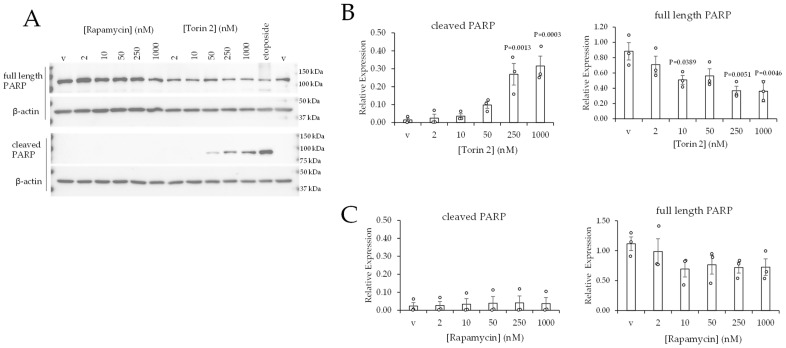
Torin 2 increases apoptosis in 786-0 cells. (**A**) Western blot data of 786-0 cells treated with vehicle, rapamycin, or Torin 2 for 24 h. (**B**,**C**) Quantification of western blot data. Data are represented as the average ± SEM of N = 3. Statistical analyses were performed using one-way ANOVA followed by Dunnett’s multiple comparison tests.

We asked whether the decrease in 786-0 cell viability in response to Torin 2 was correlated with an increase in apoptosis. The accumulation of cleaved PARP is a well-established readout of apoptosis [38]. This can be assessed by western blotting with an anti-cleaved PARP antibody. The cells (786-0) were treated with increasing (2, 10, 50, 250, and 1000 nM) concentrations of rapamycin or Torin 2 for 24 h. The results showed a statistically significant increase in the accumulation of cleaved-PARP in response to increasing concentrations of Torin 2 (Figure 6B). The increase in cleaved PARP was correlated with a significant decrease in the levels of full-length PARP in response to Torin 2 (Figure 6B). Etoposide is known to induce apoptosis in cancer cells [39], and it increased the levels of cleaved PARP in 786-0 cells as a positive control (Figure 6A). The levels of cleaved-PARP were below the level of detection in 786-0 cells treated with rapamycin (Figure 6A,C). The inability of rapamycin to induce apoptosis was anticipated, given that rapamycin did not reduce the percentage of viable 786-0 cells (Figure 5A).

Collectively, these data show that a reduction in phospho-NDRG1 (Thr346) that is greater than 90% is correlated with an increase in the apoptosis of 786-0 cells. The levels of phospho-NDRG1 (Thr346) dropped below 90% when Torin 2 was applied at 50, 250, and 1000 nM concentrations, which also stimulated the accumulation of cleaved-PARP (Figure 3B and Figure 6B) in 786-0 cells. Torin 2, when applied at 10 nM, induced an 85% reduction in phospho-NDRG1 (Thr346), which was correlated with a 35% reduction in cell viability without a correlating increase in apoptosis (Figure 3B and Figure 6B). This suggests that the residual phospho-NDRG1 (Th346) in cells treated with 10 nM Torin 2 might contribute to the prevention of apoptosis. We, therefore, hypothesize that a greater than 90% reduction in phospho-NDRG1 (Thr346) is the threshold concentration to stimulate apoptosis in 786-0 cells.

## 3. Discussion

In this study, we characterized the regulation of NDRG1 phosphorylation on Thr 346 in ccRCC. The premise for this line of investigation stemmed from protein array results showing that this phosphorylation mark showed the greatest fold increase in ccRCC relative to pair-matched non-cancerous renal tissue (Table 1). We show that RICTOR and mTOR in complex 2 promote the levels of NDRG1 protein and phosphorylation of NDRG1 (Thr346) (Figure 2 and Figure 3). Reducing RICTOR and dual inhibition of mTORC1 and mTORC2, but not selective inhibition of mTORC1, significantly reduced the levels of phospho-NDRG1 (Th346) in 786-0 cells (Figure 2, Figure 3 and Figure 4). This was associated with a decrease in cancer cell viability that was correlated with an increase in apoptosis when phospho-NDRG1 (Thr346) was reduced by greater than 90% (Figure 5 and Figure 6). Given that the loss of phospho-NDRG1 (Thr346) was correlated with an increase in cancer cell apoptosis in response to Torin 2, we hypothesize that phospho-NDRG1 (Thr346) promotes the survival of ccRCC cancer cells.

The anti-apoptotic effect of NDRG1 confers cancer cell resistance to hypoxia and anti-cancer drugs [34,40,41,42]. NDRG1 protein expression is robustly induced in cancer cells in response to hypoxia [34,40,41,42]. The induction of NDRG1 in response to hypoxia in cancer cells inhibits apoptosis and thus promotes cancer cell survival in tumors that have high levels of hypoxia [41]. In glioblastoma, alkylating anticancer drugs are less effective in cancer cells that express high levels of NDRG1 [34]. In this context, NDRG1 provides cancer cell resistance to apoptosis by stabilizing DNA repair proteins that counter the apoptotic-inducing activity of alkylating drugs in glioblastoma [34]. In hepatocellular carcinoma, siRNA-mediated reduction of NDRG1 sensitized cancer cells to doxorubicin-induced apoptosis. Regarding a potential mechanism, phospho-NDRG1 (Thr346) binds, stabilizes, and increases the activity of proteins that have functional roles in DNA repair and apoptosis pathways [34,42]. The binding of NDRG1 to these regulatory proteins requires NDRG1 to be phosphorylated at Thr346 [34]. Further, the phosphorylation of NDRG1 (Thr346) promotes NDRG1 activity [43]. In line with these studies, we show that a decrease in phospho-NDRG1 (Thr346) is correlated with an increase in apoptosis in ccRCC cells treated with increasing concentrations of Torin 2. We hypothesize this is mediated by phosphorylated NDRG1 (Thr346), which promotes the activity/and or stability of proteins in ccRCC cells that promote cell viability.

NDRG1 was previously shown to be increased in ccRCC compared with paired normal renal tissue [44,45]. We now show that phospho-NDRG1 (Thr346) is increased and that this phosphorylation is maintained by mTORC2 and RICTOR (Figure 2, Figure 3 and Figure 4). NDRG1 was identified to inhibit the proliferation of ccRCC cells in vitro and their growth as tumors in mice [44]. We show that the decrease in NDRG1 activity due to inhibition of its phosphorylation is correlated with an increase in apoptosis in ccRCC cells treated with Torin 2 (Figure 3 and Figure 6). When viewed together, these studies show that NDRG1 has at least two roles in ccRCC: it inhibits cancer cell proliferation, and when phosphorylated, it inhibits apoptosis. This suggests that ccRCC tumors that have high levels of NDRG1 might grow slower and be more resistant to apoptosis.

Our results show that Torin 2 stimulates NDRG1 mRNA expression. As mentioned, hypoxia in the tumor microenvironment stimulates NDRG1 expression in cancer cells [34,40,41,42]. This, in turn, promotes cancer cell survival [41]. Radiotherapy and alkylating drugs induce NDRG1 mRNA and protein in glioblastoma [34]. This, in turn, mediates tumor resistance to cancer therapy [34]. Di-2-pyridylketone thiosemicarbazones, which are iron chelators, stimulate an increase in NDRG1 transcription and protein expression [46,47]. This effect, in turn, has a tumor-suppressive effect on cancer cells [46,47]. The NDRG1 response to Torin 2 is different from other responses, given that increases in mRNA are not associated with an increase in total NDGR1 protein (Figure 3). This is likely due to at least two factors. First, total NDRG1 is the sum of unphosphorylated and phosphorylated NDRG1. Torin 2 induces a profound decrease in phospho-NDRG1, and this reduces a large percentage of total NDRG1 (Figure 3). Second, increases in NDRG1 mRNA must be translated into NDRG1 protein, and Torin 2, by inhibiting mTORC1, might interfere with the translation of NDRG1 mRNA into NDRG1 protein. The significant induction of NDRG1 mRNA occurred in response to high (250 and 1000 nM) concentrations of Torin 2 (Figure 3). It is possible that the loss of phospho-NDRG1 (Thr346) at these concentrations induces an increase in NDRG1 mRNA as a compensatory mechanism to restore the loss of activated NDRG1. The induction of NDRG1 mRNA could also be a response to apoptosis in cells treated with 250 and 1000 nM Torin 2 (Figure 6).

Our results suggest that RICTOR has two roles regarding the regulation of NDRG1. First, it promotes NDRG1 protein expression without changing the levels of NDRG1 mRNA (Figure 2). This suggests RICTOR promotes the stability of the NDRG1 protein. Second, RICTOR facilitates the phosphorylation of NDRG1 (Thr346) as a required component of mTORC2. This is in line with publications by other investigators that have shown RICTOR has dependent and independent mTORC2 roles [48]. We hypothesize that RICTOR could stimulate NDRG1 protein stability by associating with NDRG1 in a complex that does not contain mTOR. This could explain why reducing RICTOR, but not inhibition of mTOR reduced the levels of total NDRG1 protein (Figure 2 and Figure 3).

In conclusion, we set out to identify mTOR-phosphorylated proteins that are differentially expressed in renal tumors compared with normal renal tissue. The impetus for this question was based on genomic studies showing that approximately 20% of ccRCC cases had a mutation in at least one gene associated with the mTOR pathway [21]. The second reason to study mTOR signaling in ccRCC is that mTORC1 inhibitors are FDA-approved for the treatment of advanced ccRCC. Our proteomic array data shows the mTORC1 pathway is not statistically significantly increased in the 22 renal tumor samples we assayed relative to normal renal tissue. It is possible that a larger sample size might have detected increased mTORC1 signaling in a set of tumors. We also could not evaluate a correlation between the grade of the tumor and mTORC1 due to the sample size. We, however, did find a recurrent statistically significant increase in total NDRG1 and phospho-NDRG1 (T346) in the renal tumors that we analyzed (by western blot and protein array) (Table 1 and Figure 1). We also established a correlation between decreases in phospho-NDRG1 (Thr346) and increases in cancer cell apoptosis in cells treated with Torin 2. We propose that new therapies that selectively target the kinase that phosphorylates NDRG1 (Thr346) in renal cancer cells might be efficacious in ccRCC.

## 4. Materials and Methods

### 4.1. Reagents and Cell Culture

Immortalized human kidney epithelial cells (HK-2) were purchased from the American Type Culture Collection (ATCC, Manassas, VA, USA, Item No. CRL-2190) and were cultured according to ATCC guidelines in keratinocyte-free media with added bovine pituitary extract (50 μg/mL) and recombinant epithelial growth factor (EGF) (5 ng/mL) purchased from Fisher Scientific (Gibco, Carlsbad, CA, USA, Item No. 17005-042). The 786-0 cancer cell line is a model of clear cell renal cell carcinoma that was purchased from the ATCC and cultured in RPMI-1640 supplemented with 10% fetal bovine serum (FBS) and Penicillin–Streptomycin (P/S). The 786-0 cells were purchased from ATCC by the corresponding author and used within 15 passages. HK-2 and 786-O cells were cultured at 37 °C with 5% CO_2_. RPMI-1640, P/S, and FBS were purchased from Thermo Fisher Scientific (Waltham, MA, USA). Transient transfection was carried out with 100 nM of non-targeting short interfering RNA (siRNA) (Dharmacon, Lafayette, CO, USA, D-001810-10-05), Rictor siRNA I (Cell Signaling Technology, Danvers, MA, USA, 8649), Rictor siRNA II (Cell Signaling Technology, 8622), and Lipofectamine RNAiMAX (Thermo Fisher Scientific, 13778075) in serum-free RPMI-1640 (786-O cells) or keratinocyte-free media (HK-2 cells) for six hours, after which they were maintained in RPMI-1640 with 10% FBS (for 786-O cells) or keratinocyte-free media with bovine pituitary extract (50 µg/mL) and EGF (for HK-2 cells) for 72 to 96 h. HK2 and 786-0 cells were treated with rapamycin or Torin 2 (at 2, 10, 50, 250, and 1000 nM) in complete HK2 or 786-0 media, respectively, for 24 h for western blot and RNA studies and 96 h for cell viability studies.

### 4.2. Tissue Samples

De-identified frozen patient renal tissue samples were obtained from the biorepository bank at the Edwards Comprehensive Cancer Center at Cabell Huntington Hospital, Huntington, WV. Pathology established that renal tumors were clear-cell renal carcinoma and that pair-matched normal renal tissue was non-cancerous. De-identified samples were obtained with patient consent. Renal tissue samples were collected and immediately taken to the surgical pathology department for identification by a pathologist. Malignant and normal renal tissue were isolated using sterile techniques, with blade changes made between cutting tumor and non-tumor tissue. Isolated tissues were immediately flash-frozen in liquid nitrogen and stored in the liquid nitrogen vapor phase.

### 4.3. Reverse-Phase Protein Array

Frozen renal tissue samples were shipped on dry ice to the Reverse Phase Protein Array (RPPA) Core Facility at the University of Texas MD Anderson Cancer Center. Sample lysis and protein extraction in RPPA lysis buffer were carried out by the RPPA Core. Briefly, tissue extract was serially diluted two-fold for 5 dilutions (undiluted, 1:2, 1:4, 1:8; 1:16) and arrayed on nitrocellulose-coated slides in an 11 × 11 format to produce sample spots. Sample spots were then probed with antibodies by a tyramide-based signal amplification approach and visualized by a DAB colorimetric reaction to produce stained slides. Stained slides were scanned on a Huron TissueScope scanner to produce 16-bit tiff images. Sample spots in tiff images were identified and their densities were quantified by the Array-Pro Analyzer. Relative protein levels for each sample were determined by interpolating each dilution curve produced from the densities of the 5-dilution sample spots using a standard curve (SuperCurve) for each slide (antibody). SuperCurve is constructed by a script in R written by Bioinformatics. All relative protein level data points were normalized for protein loading and transformed to linear values.

### 4.4. Western Blot

Frozen kidney tissue samples (40 mg) were homogenized using a handheld electric tissue homogenizer (Bio-Rad, Hercules, CA, USA) in 1.0 mL of ice-cold lysis buffer (20 mM Tris-HCl (pH 7.5), 150 mM NaCl, 1 mM Na2EDTA, 1 mM EGTA, 1% Triton X, 2.5 mM sodium pyrophosphate, 1 mM β-glycerophosphate, 1 mM Na3VO4, 1 µg/mL leupeptin). Cells (in 6-well plates) were lysed in 300 µL RIPA buffer. Cell lysis and RIPA buffers were supplemented with an added mixture of protease and phosphatase inhibitors (Thermo Fisher Scientific, A32959). Tissue and cell lysates were sonicated for 30 s on ice and clarified by centrifugation for 10 min at 4 °C. Protein concentration in lysate was measured with a BCA protein assay kit (Cell Signaling Technology, 7780). Protein lysate (5 to 15 μg) was diluted in 2x laemmli sample buffer with β-mercaptoethanol, heat denatured (~100 °C for 5–8 min), separated by SDS/PAGE, and transferred to immune-blot polyvinylidene difluoride (Bio-Rad, 1620177). Blots were incubated in 5% nonfat dry milk in tris buffer saline with 0.05% Tween 20 (Bio-Rad) for 1 h and then primary antibody overnight, followed by the secondary antibody for 90 min, and antibody-antigen complexes were detected by enhanced chemiluminescence (Bio-Rad, 1705061). The ChemiDoc MP Imaging System (Bio-Rad) was used to quantify band density and acquire western blot images. Bands were analyzed for volume intensity, which is the sum of all intensities within the lane boundaries without background subtraction (image lab 4.0). Blots were stripped, and equal loading was confirmed by reprobing blots for (β-actin). Relative protein levels were quantified and normalized to β-actin. Anti-NDRG1 (#5196S), anti-phospho-NDRG1 (Thr346) (#5482), anti-β-actin (#4970), anti-Rictor (#2114), anti-S6 (#2217), anti-phospho-S6 (235/236) (#4858), anti-PARP (#9532), and anti-cleaved PARP (#5625) were purchased from Cell Signaling Technology (Danvers, MA, USA)). Antibodies were diluted for western blot application in accordance with the datasheet provided by Cell Signaling Technology.

### 4.5. Reverse Transcription and Real-Time Polymerase Chain Reaction (RT-qPCR)

Total RNA was isolated from cells in 6-well plates with RNeasy Plus kits (Qiagen, Germantown, MD, USA, #74134) and reverse transcribed using cDNA synthesis kits (Applied Biosystems (Foster City, CA, USA)). Real-time PCR was conducted with StepOnePlus (Applied Biosystems) and SYBR Green master mix (Applied Biosystems) in accordance with the suppliers’ protocols. Samples were run in triplicate. Relative mRNA levels were quantified and normalized to GAPDH or β-actin. Relative changes in gene expression were quantitated using the 2−ΔΔCT formula. Primers for GAPDH forward 5′-CATGAGAAGTATGACAACAGCCT-3′, reverse 5′-AGTCCTTCCACGATACCAAAGT-3′, Rictor forward 5′-GACTGAAACCCGTCAATATGGC-3′, reverse 5′-TGTCATTCCGCCCTCGTACT-3′, NDRG1 forward 5′-AGCTCGTCAGTTCACCATCC-3′, reverse 5′-GAGTACGCGGGGCTACAAAG-3′, beta-actin forward 5′-CTCGCCTTTGCCGATCC-3′, reverse 5′-TCTCCATGTCGTCCCAGTTG-3′ primers were purchased from Sigma-Aldrich (St. Louis, MO, USA).

### 4.6. Live–Dead Assays

Cells were seeded in quadruplicate in 96-well plates at 5000 cells/well and treated the following day with vehicle, rapamycin, or Torin 2, and the % of viable cells was determined by calcein-AM (2 µM) fluorescence (ex/em 495 nM/515 nM) and calculated relative to the vehicle group. The cells (786-0) were transfected with siRNAs (100 nM) and plated in 96-well plates at 5000 cells/well. HK2 cells were plated in 96-well plates and, the following day, transfected with siRNAs (100 nM). At 96 h post-transfection, the % of viable cells was determined by calcein-AM fluorescence and calculated relative to the control siRNA group. Calcein-AM was purchased from Thermo Fisher Scientific (L3224). Fluorescence was measured by a 96-well plate reader.

### 4.7. Statistical Methods

For RPPA (N = 22), log2 fold changes were determined by taking the log2 expression in the tumor minus the log2 expression in the normal for each patient and then taking the mean of those. Paired *t*-tests were conducted for each protein and phospho-protein, and then Benjamini–Hochberg was used to calculate adjusted *p*-values (false discovery rates) to account for multiple hypothesis testing. Differences between tumor and normal tissue protein expression from quantified western blot data were analyzed using paired *t*-tests using GraphPad Prism 9. Statistical analyses were performed using a two-tailed *t*-test or one-way ANOVA, followed by Dunnett’s multiple comparisons test for the remaining in vitro studies. In vitro data are represented as the means with SEM of three independent experiments. Cell viability data are indicated as the means with SD of four replicates.

## Figures and Tables

**Table 1:** Differentially expressed proteins and phospho-proteins in ccRCC samples relative to pair-matched normal renal tissue (N = 22).

Protein	Fold Change	FDR (BH) ^1^
Mitochondrially Encoded Cytochrome C Oxidase I	5.861 Decreased	0.0002079
L1 Cell Adhesion Molecule	5.786 Decreased	0.0004514
Hexokinase 2	4.826 Increased	0.0012272
Mucin 1, Cell Surface Associated	3.37 Decreased	0.0002613
phospho-N-Myc Downstream Regulated 1 (Thr346)	3.298 Increased	0.0027587
phospho-Glycogen Synthase 1 (S641)	3.189 Increased	0.0013911
UDP Glucuronosyltransferase Family 1 Member A Complex	2.79 Decreased	0.0007309
Succinate Dehydrogenase Complex Iron–Sulfur Subunit B	2.603 Decreased	0.0003587
Cadherin 1	2.516 Decreased	0.0000798
Claudin 7	2.336 Decreased	0.0007309
Cyclophilin D	2.318 Decreased	0.0007309
Pyruvate Kinase M1/2	2.285 Increased	0.0082690
Lactate Dehydrogenase A	2.122 Increased	0.0067436
Cytochrome C Oxidase Subunit 4I1	2.11 Decreased	0.0005251
Glycogen Synthase 1	2.076 Increased	0.0066912
Ladinin 1	1.973 Decreased	0.0010417
Ubiquinol-Cytochrome C Reductase Core Protein 2	1.907 Decreased	0.0005251
Glutaminase	1.904 Decreased	0.0007309
RAB25, Member RAS Oncogene Family	1.868 Decreased	0.0002079
Succinate Dehydrogenase Complex Flavoprotein Subunit A	1.851 Decreased	0.0008878
Heat Shock Protein Family A (Hsp70) Member 1A	1.833 Increased	0.0021894
Heat Shock Factor Binding Protein 1	1.81 Increased	0.0028349
Tyrosine 3-Monooxygenase/Tryptophan 5-Monooxygenase Activation Protein Epsilon	1.8 Decreased	0.0005230
Carbonic Anhydrase 9	1.798 Increased	0.0013911
ATP Synthase F1 Subunit Alpha	1.798 Decreased	0.0006969
phospho-Proline-Rich Akt Substrate, 40 Kd (Thr346)	1.732 Increased	0.0008642
Histone-H3	1.729 Increased	0.0064733
Glutamate Dehydrogenase 1	1.72 Decreased	0.0020744
Mitofusin 2	1.702 Decreased	0.0005251
Gap Junction Protein Alpha 1	1.672 Increased	0.0008768
Indoleamine 2,3-Dioxygenase 1	1.67 Increased	0.0327598
phospho-p44/42 MAPK (Erk1/2) (Thr202/Tyr204)	1.645 Increased	0.0075331
H2B Clustered Histone 3	1.642 Increased	0.0028756
Solute Carrier Family 16 Member 3	1.625 Increased	0.0030439
Serpin Family E Member 1	1.605 Increased	0.0266041
Dipeptidyl Peptidase 4	1.571 Decreased	0.0050791
Autophagy Related 7	1.561 Decreased	0.0006969
Phosphoglycerate Dehydrogenase	1.553 Decreased	0.0029476
XPA, DNA Damage Recognition and Repair Factor	1.549 Increased	0.0019721
Fibronectin 1	1.545 Increased	0.0166762
Transcription Factor A, Mitochondrial	1.532 Decreased	0.0004514
Phospho-Glycogen Synthase Kinase 3 alpha/beta (Ser21/9)	1.523 Increased	0.0003587
Solute Carrier Family 1 Member 5	1.516 Decreased	0.0020998
Vav Guanine Nucleotide Exchange Factor 1	1.516 Increased	0.0180443
Protein Tyrosine Kinase 2 Beta	1.508 Decreased	0.0006910
Dual Specificity Phosphatase 4	1.502 Decreased	0.0006005
phospho-Yes1 Associated Transcriptional Regulator (S127)	1.5 Increased	0.0098237

^1^ False Discovery Rate (FDR), calculated by the Benjamini–Hochberg (BH) method.

## Data Availability

Not available.
